# Effects of Mild Cold Shock (25°C) Followed by Warming Up at 37°C on the Cellular Stress Response

**DOI:** 10.1371/journal.pone.0069687

**Published:** 2013-07-23

**Authors:** Thibaut Neutelings, Charles A. Lambert, Betty V. Nusgens, Alain C. Colige

**Affiliations:** Laboratory of Connective Tissue Biology, Interdisciplinary Grouping of Applied Genoproteomic - Research, University of Liège, Liège, Belgium; German Cancer Research Center, Germany

## Abstract

Temperature variations in cells, tissues and organs may occur in a number of circumstances. We report here that reducing temperature of cells in culture to 25°C for 5 days followed by a rewarming to 37°C affects cell biology and induces a cellular stress response. Cell proliferation was almost arrested during mild hypothermia and not restored upon returning to 37°C. The expression of cold shock genes, CIRBP and RBM3, was increased at 25°C and returned to basal level upon rewarming while that of heat shock protein HSP70 was inversely regulated. An activation of pro-apoptotic pathways was evidenced by FACS analysis and increased Bax/Bcl2 and BclX_S/L_ ratios. Concomitant increased expression of the autophagosome-associated protein LC3II and AKT phosphorylation suggested a simultaneous activation of autophagy and pro-survival pathways. However, a large proportion of cells were dying 24 hours after rewarming. The occurrence of DNA damage was evidenced by the increased phosphorylation of p53 and H2AX, a hallmark of DNA breaks. The latter process, as well as apoptosis, was strongly reduced by the radical oxygen species (ROS) scavenger, N-acetylcysteine, indicating a causal relationship between ROS, DNA damage and cell death during mild cold shock and rewarming. These data bring new insights into the potential deleterious effects of mild hypothermia and rewarming used in various research and therapeutical fields.

## Introduction

While heat shock has been intensively investigated, cold shock has retained relatively less attention. Cooling at various temperatures and subsequent rewarming however happen even for homeothermic animals or cultured cells, tissues and organs in a number of physiological or accidental situations. Hypothermia is largely used during cardiac surgery or treatment of brain damage. Preservation and transportation of organs and cells usually take place at low temperature, and the production of recombinant proteins is improved by lowering temperature [1–4]). The return to normothermia after cooling induces at least some of the phenotypical changes observed upon hyperthermia, suggesting that cells somehow acclimatize to mild temperature and sense a relative, rather than an absolute, hypothermia [5].

Mild cold stress (25-35°C) and heat shock induce somehow similar phenotypical modifications. A general decrease of transcription and translation rate has been reported, although the expression of a subset of temperature-sensitive proteins is not modified or even increased [6,7]. Regulations affecting mRNA stability, alternative transcription start site and splicing decisions have also been documented [8,9]. Reduced metabolism [10,11], cell cycle arrest [12], activation of apoptotic program, disassembly of the cytoskeleton and altered composition or fluidity of lipidic membranes have been reported [4,11,13]. Contrasting to these features common to both temperature shifts, heat shock can also induce autophagy, a process protecting cells from death [14–16]. Hypothermia has been reported to reduce the level of intracellular reactive oxygen species (ROS) while hyperthermia would stimulate their production [17].

Among a significant number of described cold shock proteins (CSP), only CIRBP (cold-inducible RNA binding protein) and RBM3 (RNA binding motif protein 3), two highly homologous proteins, have been thoroughly characterized. Their expression is rapidly and markedly increased during mild hypothermia, and they appear to be key determinants in cold-stress adaptation and to stimulate translation of cold-specific transcripts through various mechanisms [7,11,18]. CIRBP mRNA expression is transcriptionally controlled via cold-responsive elements in its promoter [4]. The study of the regulation of the cell cycle by CIRBP has given conflicting data. Cells overexpressing CIRBP at 37°C have a reduced growth rate and are mainly arrested in G1 phase [19]. Contrasting to these data, overexpression of CIRBP was reported to immortalize mouse embryo fibroblasts while its down-regulation decreased cell proliferation [20]. RMB3 is also associated with proliferation and considered as a proto-oncogene ( [21], and references therein).

The HSP are induced upon heat-shock or upon return to normothermia after a cold shock. Some HSP act as proteins chaperone, preventing misagreggation of denatured proteins and assisting correct refolding upon return to normothermia, while others regulate protein turnover. HSP27, HSP60, HSP70 and HSP90 are generally recognized to prevent apoptosis although some pro-death effects have been described. Some HSP also regulate the redox state of the cells [22].

Our laboratory is involved in space research and investigates the effect of weightlessness on cells phenotype during space flights in autonomous capsules or on the ISS (for detailed descriptions of the experiments, see Erasmus Experiment Archive database from ESA at http://eea.spaceflight.esa.int). The usual experimental time schedule implies a delay of several days between the preparation of the cell cultures on Earth, their transportation to the site of take off (Cape Canaveral, Baikonour...), integration in the space vessel, launch, orbiting and transfer to the ISS. During this period, cells are usually kept at 22-27°C, considered to provide a “sleeping mode” avoiding disturbances due to vibrations and short periods of hypergravity during launch [23]. Thereafter, cultures are transferred to an incubator at 37°C for experiment in microgravity. During preparatory experiments, we observed that morphology was affected by rewarming cells at 37°C after several days at 25°C. These observations suggested the induction of a cellular stress that might represent a confounding bias in the interpretation of the microgravity data. We therefore thought to investigate the phenotype of cells during storage at 25°C followed by rewarming at 37°C. These data bring new cellular and molecular mechanisms that might benefit to research and therapeutical fields using hypothermia. They will further allow to set up a more appropriate experimental design for future space experiments.

## Materials and Methods

### Cell culture and proliferation assays

WI26 cells (SV40 transformed human lung fibroblasts, ATCC: CCL-95.1), MG-63 (human osteosarcoma cell line, ATCC: CRL-1427), HeLa (epithelial containing HPV cell line, CRM-CCL- 2), HMEC (human dermal microvascular endothelial cells, ATCC: CRL-10636) and HBME-1 (SV40 immortalized human bone marrow endothelial cells [24]) were routinely grown at 37°C in Dulbecco’s Modified Eagle’s Medium (DMEM, Lonza, Verviers, Belgium) buffered with sodium bicarbonate and supplemented with 10% Foetal Bovine Serum (Lonza), 100 U/ml penicillin/ streptomycin (Lonza) under 5% CO_2_. For cold shock and rewarming experiments, cells were trypsinized, seeded at 10,000 cells/cm^2^ and cultured for 24h at 37°C. Samples were then collected and frozen at -80°C prior to protein or RNA extraction and constituted the control group (T0). For mild hypothermia experiments, culture medium was replaced with the same medium supplemented by 25 mM HEPES and cultures were maintained for 1 to 5 days at 25°C and then transferred to 37°C for 1 to 24 hours as indicated.

Cells number was evaluated by quantifying DNA using bis-benzimide (Hoechst GmbH, Francfort, Germany) and fluorimetry at 355 nm excitation and 460 nm emission wavelengths using a SpectraMax Gemini XS apparatus (MDS Analytical Technologies, Sunnyvale, CA, USA) as described [25]. Cell proliferation rate was measured by [^3^H]-thymidine incorporation. WI26 cells (4x10^4^) were seeded and cultured overnight in multidishes 4 wells plates in DMEM. After renewing the culture medium by DMEM supplemented with HEPES, cells were maintained at 25°C for 4 days. [^3^H]-thymidine (1µM final concentration, 2.5 Ci/mol, Perkin Helmer, Waltham, MA, USA) was then added to the culture medium. After the indicated times of culture at 25°C and 37°C, 10% TCA-insoluble radioactivity was measured by liquid scintillation radioluminescence using Aqualuma plus (Lumac LSC BV, Groningen, the Netherlands) and a β-counter (TriCarb 1900 TR, Packard BioScience, Vic, Australia).

### Western-Blot analysis

Antibodies against JNK (#9252), phospho-JNK (#92515), AKT (#9272), phospho-AKT (#9271), phospho-p53 (#9284), H2AX (#9718) and phospho-histone H2AX (#9718) were purchased from Cell Signaling Technology, Inc. (Beverly, MA, USA), anti-p53 (#sc-6243) from Santa Cruz (Santa Cruz, CA, USA), anti-LC3 (#PM034) from MBL (Naka-ku Nagoya, Japan), anti-MAP kinase (p42/44) (# M-5670) and anti-phospho-MAP kinase (#M8159) from Sigma-Aldrich (St-Louis, MO, USA), GAPDH (#MAB374) from Millipore (Temecula, CA, USA). Secondary horseradish peroxydase-conjugated antibodies (rabbit anti-mouse IgG P0260 and swine anti-rabbit P0217) were from DAKO (Glostrup, Denmark). Cells were lysed in Laemmli buffer supplemented with 50 mM dithiothreitol (DTT). Proteins were run on a 15% acrylamide gel and transferred to PVDF transfer membrane (NEN Life Science Products, Boston, MA, USA) at 20V overnight. Membranes were blocked for 1 hour with 3% dry milk in PBS-Tween (0.05% Tween 20 in PBS), incubated for 2 hours or overnight with the primary antibody, washed three times with PBS-Tween and incubated for one hour in horseradish peroxydase-conjugated secondary antibody. After washing with PBS-Tween, immunoreactivity was revealed by chemoluminescence using an ECL kit (Amersham Biosciences, Buckinghamshire, UK) and X-ray film exposure. To control proteins loading on the gels, the membranes were further probed with extracellular signal-regulated kinase p42/44 (MAPK) (Erk1/2) or GAPDH antibodies.

### Analysis of mRNA expression

Total RNA was prepared using High Pure RNA Isolation Kit (Roche Molecular Biochemicals, Branchburg, NJ, USA) according to the manufacturer’s recommendations and quantified by spectrophotometry (NanoDrop ND-1000, Isogene Life science, IJsselstein, The Netherlands). 10 ng of total RNA were reverse transcribed and amplified using GeneAmp Thermostable rTth Reverse Transcriptase RNA PCR Kit (Perkin-Helmer, Boston, MA, USA) and specific pairs of primers (Eurogentec, Seraing, Belgium) in an automated thermal cycler (GeneAmp PCR System 2400 or 9600, PerkinElmer, Norwalk, CT, USA). The RT step was at 70°C for 15 minutes. Denaturation of RNA/DNA heteroduplexes for 2 minutes incubation at 95°C was followed by PCR amplification for adequate number of cycles (25 to 35) and a final elongation step of 2 minutes at 72°C. The PCR conditions for amplification of the various genes were 15s of denaturation at 94°C; 20s of primer annealing at 66°C and 10s of polymerization at 72°C, except for HSP70: 15s at 95°; 30s at 60° and 30s at 72°. The primers sequence, number of PCR cycles and size of the expected RT-PCR products are described in Table S1. Primer pairs allow the RT-PCR amplification of all known splice variants of Bcl-X, BAX and VEGF-A mRNA and their discrimination by electrophoresis on the basis of the size of their amplification products.

The PCR products were analyzed by electrophoresis on 10% polyacrylamide. After staining with GelStar dye (FCM Bioproducts, Rockland, ME, USA), the signals were quantified using Fluor-S Multimager and the software Quantity One 4.6 (BioRad, Hercules, CA, USA). Each RT-PCR experiment included a no template control which showed no signal. The results were expressed in arbitrary units per unit of 28S rRNA used as calibrator. For the measurement of 28S rRNA a synthetic RNA (28S Ctrl) reverse transcribed and amplified with the same primers as the cellular RNA was added in each reaction tube to monitor reaction efficiencies [26,27].

### Cell survival and apoptosis

Cell survival was quantified by trypan blue exclusion assay. Adherent cells were detached with 2.5% trypsin-EDTA and added to the culture supernatant to take account of the floating cells. Cells were pelleted and suspended in 0.5% trypan blue (BDH Chemicals Ltd, Poole, England) in PBS. Dead and viable cells were counted on a Thoma cell counting chamber (Marienfeld, Germany).

Apoptosis was evaluated by fluorescence-activated cell sorting after annexin V–FITC and propidium iodide staining. Adherent cells were detached with 2.5% trypsin-EDTA and added to the culture supernatant. Cells were pelleted, suspended in Annexin binding buffer (Annexin V-FITC Apoptosis Detection kit, Sigma) and incubated for 10 min with Annexin V-FITC (270 ng/ml) and propidium iodide (1.1 µg/ml). Flow cytometry was performed on a FACSCanto II double LASER flow cytometer (UV, 488 nm, 633 nm) (BD Biosciences) and data were analyzed using FACSDiva Software (BD Biosciences).

### ROS Measurements

The accumulation of intracellular ROS was determined by measuring fluorescence after dichlorofluorescein (DCF) loading [28]. Cells (10,000/well of 96 wells multidish) were incubated in PBS containing 25µM of 2′, 7′-dichlorodihydrofluorescein diacetate (H_2_DCF) (Sigma-Aldrich, St. Louis, Mo, USA) for 2h at 37°C. The fluorescence emitted by the oxidized dye was measured in a spectrometer SpectraMax Gemini XS apparatus at 485 nm excitation and 530 nm emission wavelengths. When indicated, 15mM of N-Acetylcysteine (Sigma-Aldrich, St. Louis, Mo, USA), was added to cultures.

### Immunofluorescence staining

Cells fixed with 3% paraformaldehyde in PBS were permeabilized with 0.1% Triton X-100 in PBS for 3 minutes and incubated in normal goat serum 1/40 in PBS for 20 minutes. They were probed with anti-LC3 or anti γH2AX (Ser139) antibodies (1/500 in PBS) for 1 hour, washed with PBS and revealed with Alexa Fluor 488-conjugated goat anti-rabbit IgG (Molecular Probes, Eugene, Oregon, USA) (1/1000 in PBS) for 1 hour. Fibrillar actin and nuclei were labeled with 1/200 TRITC-conjugated phalloidin (Sigma-Aldrich, St-Louis, MO, USA) and 1/1000 bis-benzimide (Hoechst GmbH, Francfort, Germany), respectively, for 20 minutes. After extensive washing in PBS the coverslips were mounted on histoslides (Labonord, France) and observed by inverted fluorescence microscopy (Zeiss Axiovert 25, Carl Zeiss Co., Oberkochen, Germany) with single-channel excitation and photographed using a CCD camera.

### Statistical analysis

The results are expressed as the mean values ± standard deviation. The statistical analysis was performed using the t-test of Student. The experiments were made in triplicate except otherwise indicated. Significant modulations are indicated by *p<0.05; **p<0.01; ***p<0.001, versus T0.

## Results

### 1: Mild hypothermia and rewarming affect cell morphology, proliferation, survival and expression of temperature-dependent genes

**Figure 1:**
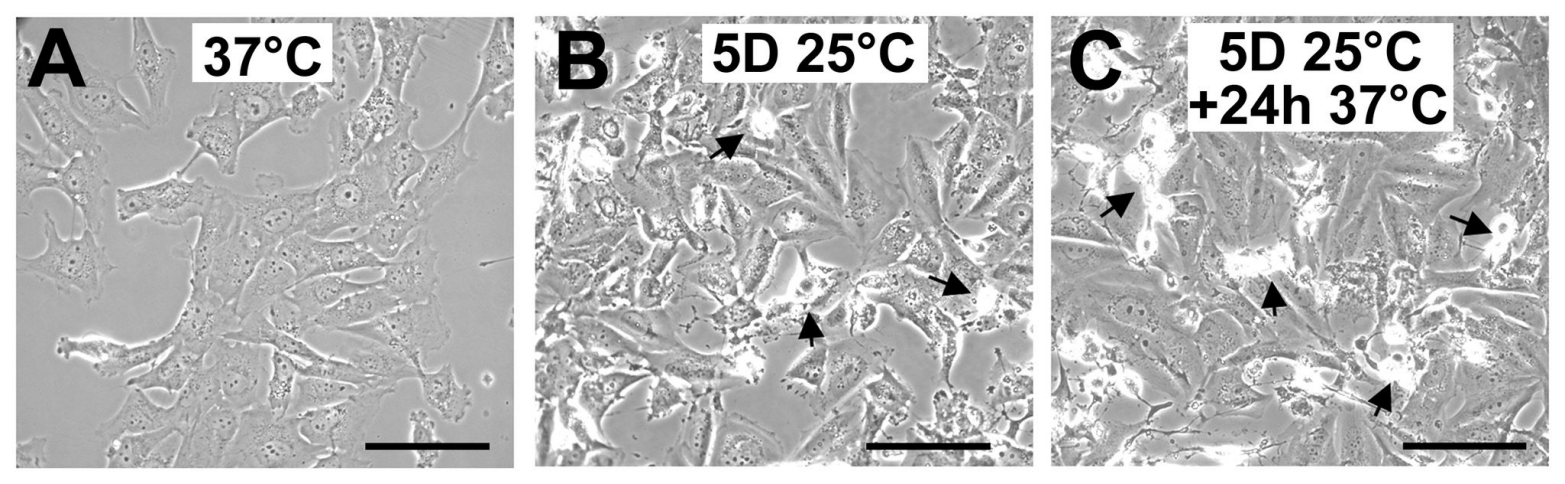
Phase contrast micrographs of WI26 cells. Cells were continuously cultured at 37°C (A), at 25°C for 5 days (B) or at 25°C for 5 days followed by a rewarming to 37°C for 24h (C). Arrows point to refringent, apoptotic-like cells. Bar: 100µm.

**Figure 2:**
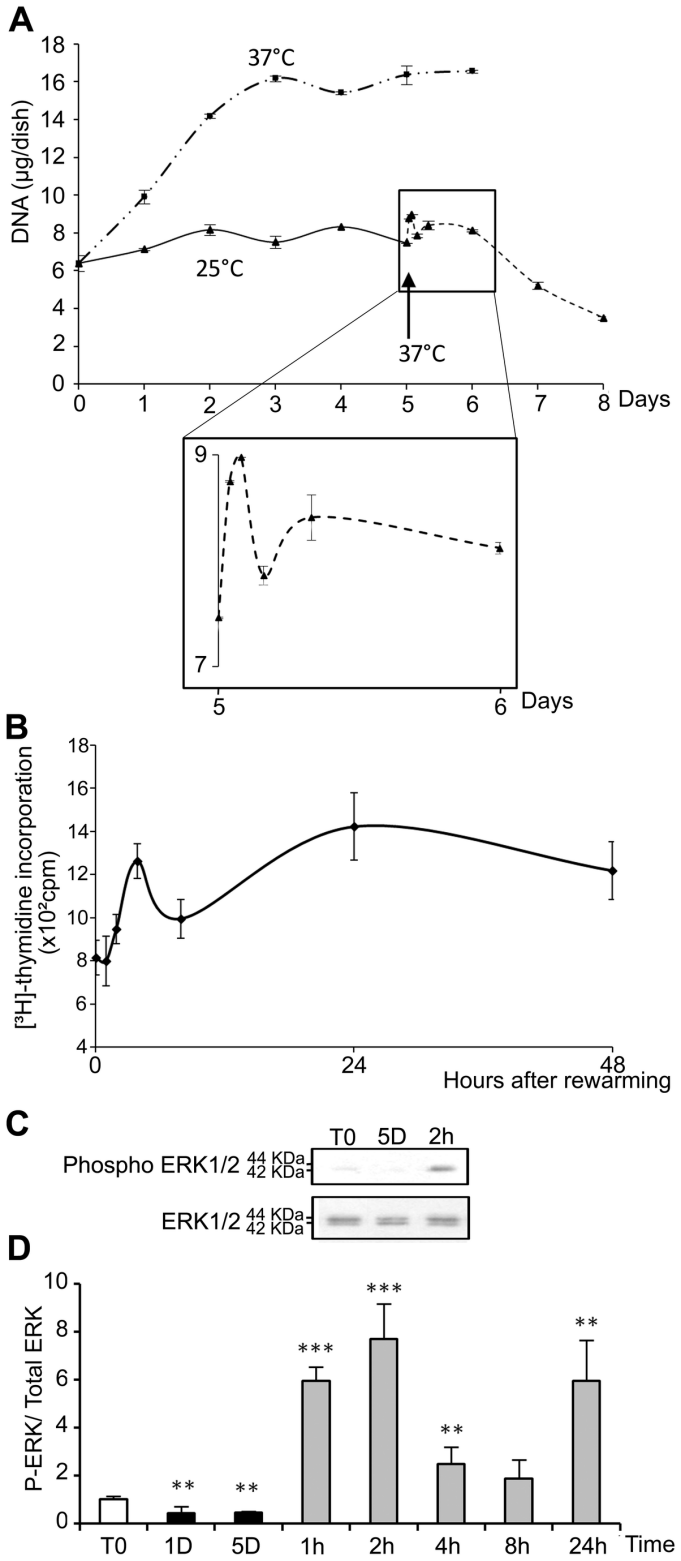
Temperature dependence of cell proliferation. (A) WI26 cells maintained at 37°C for 1 to 6 days (— - - —) or at 25°C for 1 to 5 days (——) and then warmed at 37°C for 1 to 72 hours (- - - -), and DNA content was measured. The black arrow indicates the transition from 25°C to 37°C. Insert provides an enlarged view of the first 24 hours of rewarming at 37°C. (B) [^3^H]-thymidine incorporation by WI26 cells maintained for 5 days at 25°C and warmed at 37°C for 1 to 48 h. (C) Representative western blot probed with antibodies specific for phospho ERK 1/2 or total ERK 1/2. (D) Quantification of the western blots. Data are expressed as the mean ratio of P-ERK/ total ERK normalized to ratio in control cells (T0), taken as 1. T0: 1 day at 37°C; 1D and 5D: 1 or 5 days at 25°C; 1h, 2h, 4h, 8h and 24h: 5 days at 25°C followed by 1 to 24h at 37°C.

**Figure 3:**
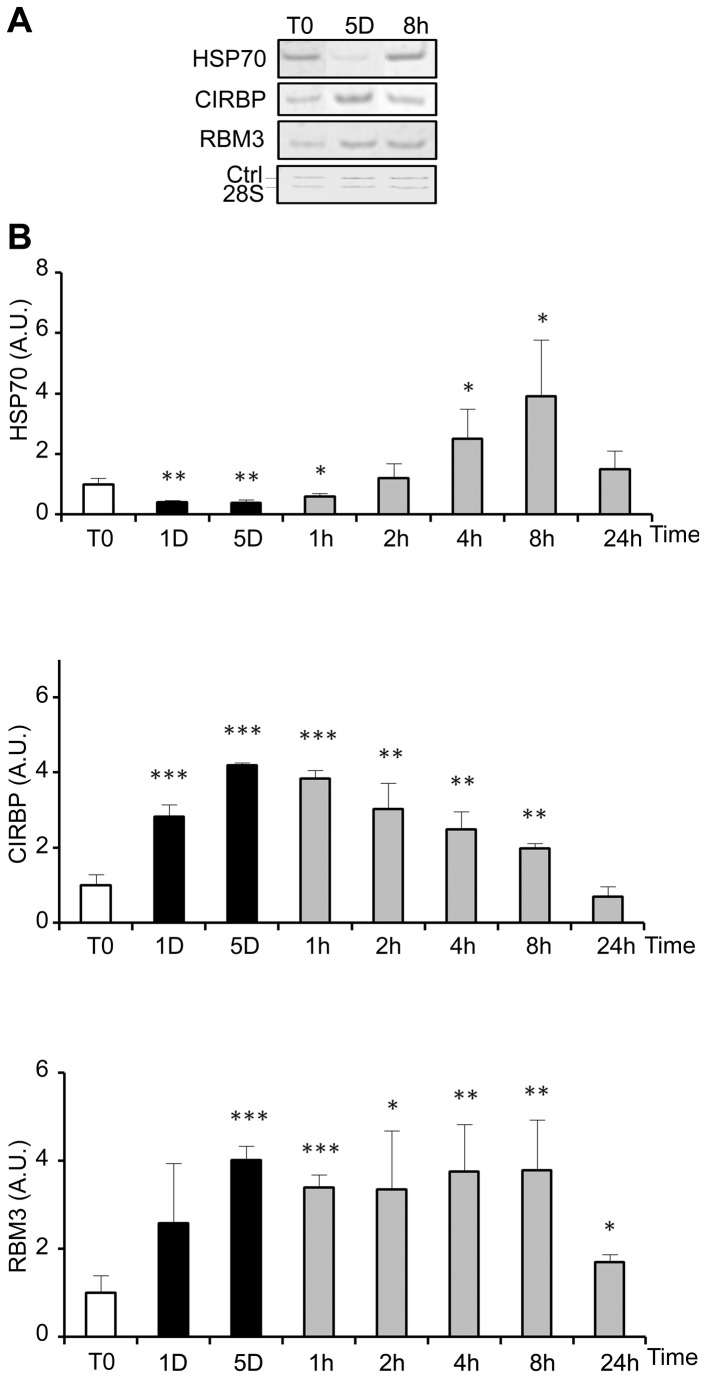
Cold-shock and rewarming affect heat and cold shock genes expression. The expression of heat shock (HSP70) and cold shock genes (CIRBP and RBM3) was quantified at the mRNA level by RT-PCR in WI26 cells cultured at 25°C for 1 and 5 days before warming up at 37°C for 1h to 24h. (A) Representative gels showing the RT-PCR amplification products. (B) HSP70, CIRBP and RBM3 mRNA levels are expressed as mean ± SD (n=3) after normalization to the 28S rRNA content used as calibrator. Values at T0 were arbitrary taken as 1. Legend for culture schedule is the same as in Figure 2.

As compared to cells kept at 37°C (Figure 1A), morphological alterations were observed in WI26 cells after 5 days of storage at 25°C (Figure 1B). After warming-up at 37°C, cells did not recover a normal morphology after 24h (Figure 1C). Instead, some were rounded, refringent and detached, suggesting induction of apoptosis. At 25°C, the number of cells as measured as the DNA content remained constant during the 5 days with no observed significant loss (Figure 2A). Upon rewarming after 5 days at 25°C, DNA content in the cultures slightly and transiently increased to drop down thereafter (insert in Figure 2A). A similar wave-shaped curve was observed for [^3^H]-thymidine incorporation, although with a slight delay (Figure 2B). Phosphorylation of ERK1/2, known to be involved in cell proliferation and survival, paralleled the proliferation curve at least up to 8h after rewarming. It was reduced during storage at 25°C and induced up to 6-8 fold 1 and 2h after rewarming (Figure 2C and D), time points corresponding to the transient DNA synthesis. The expression of HSP70, RMB3 and CIRBP was investigated at the mRNA level by RT-PCR (Figure 3). While HSP70 mRNA level was reduced at 25°C, rewarming at 37°C resulted in a progressive increase (up to 4 fold after 8h) followed by a decline. RBM3 and CIRBP mRNA levels were both increased at 25°C already at day 1 and returned close to control levels after 24h at 37°C.

**Figure 4:**
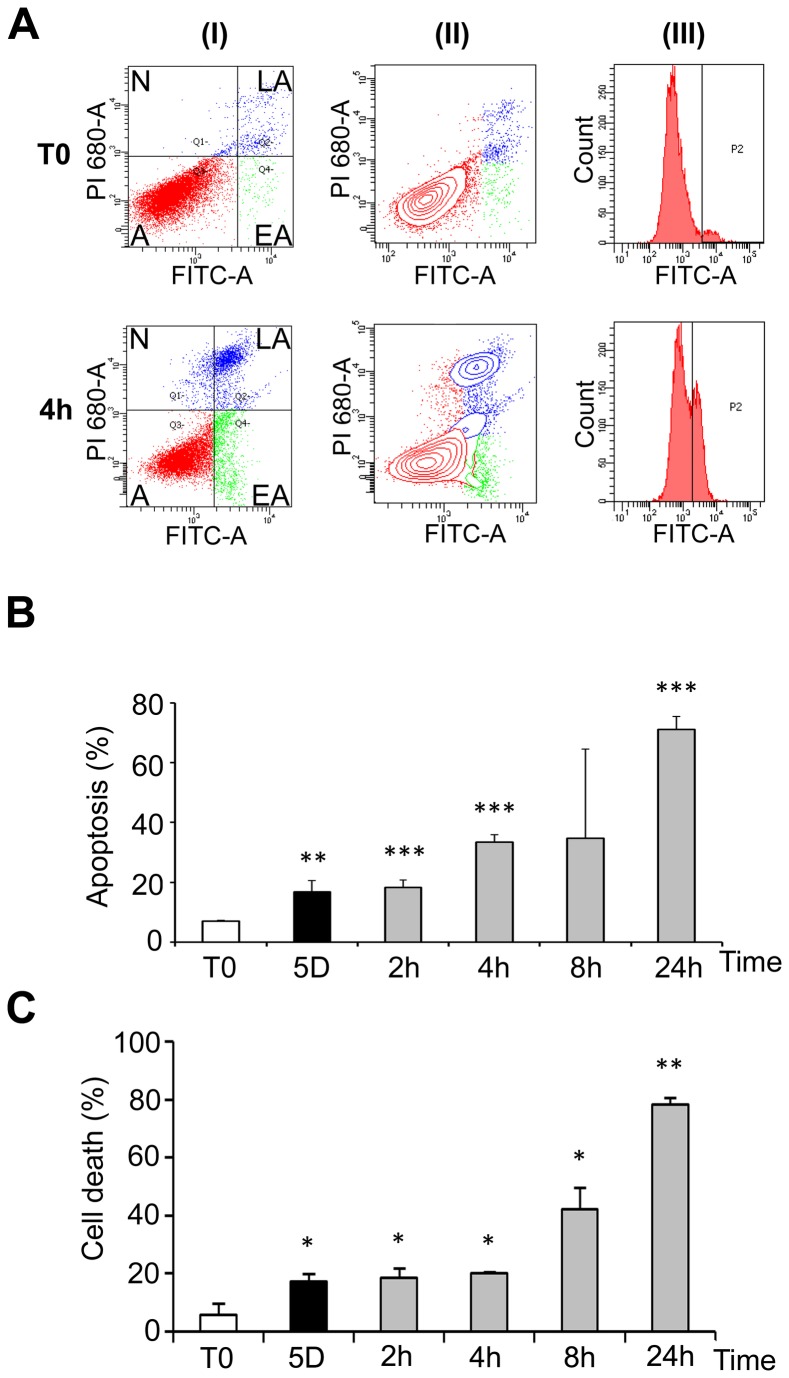
Cold shock and rewarming affect cell viability. WI26 cells were cultured at 25°C for 5 days and then warmed-up at 37°C for 2 to 24h. Cells kept at 37°C were used as control (T0). FACS analysis was performed after labeling with FITC-annexin V (FITC-A, X-axes) and propidium iodide (PI, Y-axes). 12.000 to 18.000 events were collected for each experiment. (A) Example of dots graphs (I), contour graphs (II) and annexin V curves (III) of control cells and cells maintained 5 days at 25°C and then warmed-up at 37°C for 4h (4h). Alive cells [A] (double negative staining), cells in early apoptosis [EA] (annexin V positive, PI negative), in late apoptosis [LA] (double positive) and necrotic [N] (annexin V negative, PI positive) are indicated on the graphs. Annexin V curves (III) were used to define the gating allowing to discriminate the populations. (B) Percentage of apoptotic cells as measured by FACS analysis. Cells were cultured at 37°C (T0) or 25°C for 5 days and subsequent warming-up at 37°C for 2 to 24h. (C) Percentage of dead cells as measured by trypan blue exclusion assay. Cells were cultured in duplicate for 5 days at 25°C and then rewarmed for 2 to 24h at 37°C.

To support the induction of apoptosis suggested by the morphological alterations illustrated in Figure 1, the expression of several pro- and anti-apoptotic factors was investigated at the mRNA level and the phosphorylation of Akt was evaluated by western blot. The expression of both the anti-apoptotic factor Bcl-2 and the pro-apoptotic Bcl-2-associated X protein (BAX) was decreased at 25°C and remained low for several hours after warming-up before returning to control level (Figure S1, A, B and C). However, the BAX to Bcl-2 ratio, considered as indicative of apoptosis, was increased during cold shock and early rewarming (Figure S1, D). Bcl-X exists under 2 alternatively spliced isoforms: a short pro-apoptotic form (Bcl-X_S_) and a long anti-apoptotic transcript (Bcl-X_L_). Total Bcl-X mRNA level was largely increased by cold shock and rewarming (Figure S2, B). A shift towards the short pro-apoptotic isoform, illustrated by the Bcl-X_S/L_ ratio, was observed at 25°C, and up to 8 hours after transition to 37°C (Figure S2, C). Phosphorylation of Akt, a kinase involved in survival pathways by inhibiting apoptotic processes, was first decreased after 1 day at 25°C, largely increased above control levels after 5 days at 25°C and remained elevated after warming-up (Figure S3, A and B). Together these data indicate that both pro- and anti-apoptotic processes are triggered during hypothermia and warming-up. Cells were however ultimately committed to apoptosis as shown by FACS analysis (Figure 4A and B) and trypan blue exclusion (Figure 4C). FACS data showed that a significant apoptosis was induced after five days at 25°C and more dramatically upon rewarming (Figure 4B, grey bars). Similar observations were made by measuring cell death using trypan blue exclusion test (Figure 4C). A similar induction of apoptosis was observed in osteoblastic MG-63 cells (Figure S4) suggesting that apoptosis is a general cellular response to hypothermia and rewarming.

### 2: Mild hypothermia and rewarming induce autophagy

**Figure 5:**
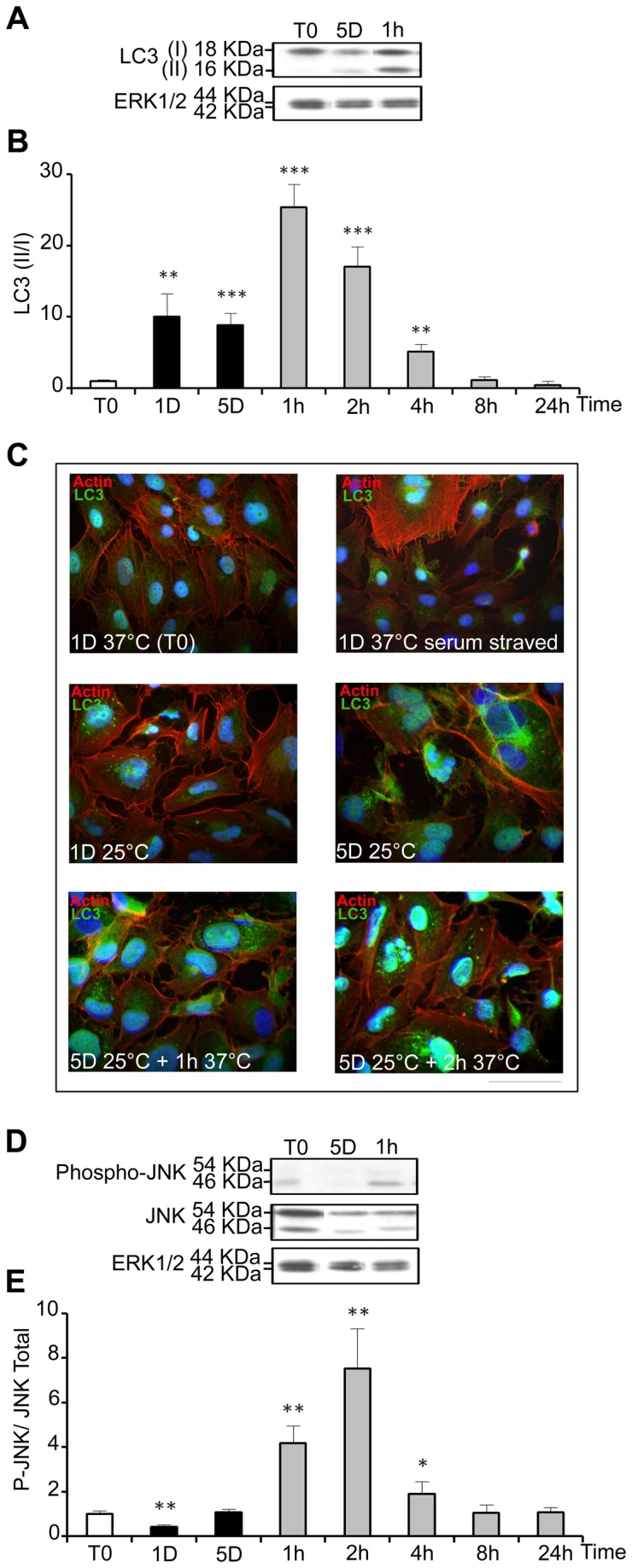
Cold shock and rewarming induce autophagy and a cellular stress response. The level of LC3 I and II (A), phospho-JNK (P-JNK) and total JNK (D) was analyzed by western blot in cells cultured at 25°C for 1 and 5 days and then warmed-up at 37°C for 1 to 24h. The levels of ERK1/2 were taken as calibrator and used to monitor protein loading. Results are expressed as the mean ratio of LC3 II/I (B) and of P-JNK/ total JNK (D) taking T0 as 1. (C) Immunostaining of LC3 was performed in WI26 cells maintained at 37°C in the presence or in the absence of serum or at various time points during the cold shock and rewarming. Actin stress fibers appear in red, LC3 in green and nucleus in blue. Bar: 100µm.

The microtubule-associated protein 1 light chain 3 (LC3) is present in cells as two isoforms: LC3 I (18kDa) located in the cytoplasm, and LC3 II (16kDa) associated with the inner membrane of autophagosomes [29], the LC3 II/I ratio being correlated to the level of autophagy. It was measured by western-blot in WI26 cells experiencing mild cold shock and warming-up (Figure 5A). LC3 II/I ratio was low in control samples, increased up to 10 fold after 1 and 5 days at 25°C and further transiently increased, up to 25 fold, during rewarming (Figure 5B). Intracellular localization of LC3 was visualized by immunostaining. Serum-starved cells as positive and cells kept at 37°C in the presence of serum were used as positive and negative controls, respectively. A clear punctuated labeling typical of autophagosomes vesicles was seen in serum-starved cells and in cells cultured for 1 and 5 days at 25°C, and was even more evident after warming-up to 37°C for 1 and 2 hours (Figure 5C). Together, these data indicate that autophagy is triggered by both hypothermia and relative hyperthermia.

Jun N-terminal kinase (JNK) activation, known to be induced by cellular stress and to mediate autophagy [30], was transiently increased after warming-up to 37°C for 1, 2 and 4 hours (Figure 5D and E). However, it was not increased after 1 day and 5 days at 25°C, suggesting that it might participe in the autophagy induced by warming up but not by cold shock.

### 3: Mild cold shock and rewarming induce ROS production and DNA damage

**Figure 6:**
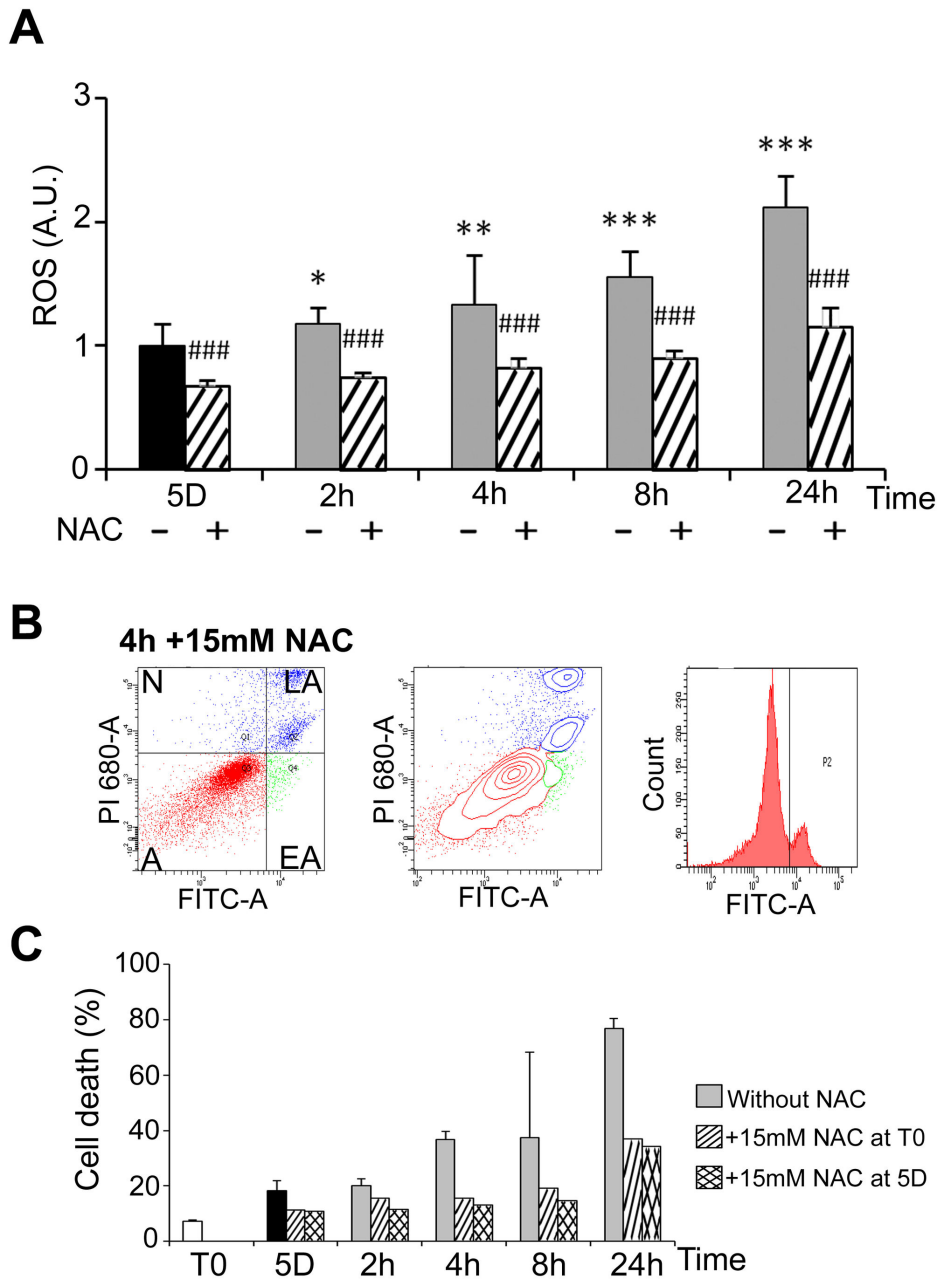
Rewarming-induced ROS production and **ROS-dependant apoptosis.** (A) ROS were measured in WI26 cells cultured at 25°C for 5 days and then warmed-up at 37°C for 2 to 24h in absence (-) and in presence (+) of 15mM N-Acetylcysteine (NAC). Data are expressed in arbitrary units taking T0 as 1. Significant inhibition by NAC versus condition without NAC is indicated # # # (p<0.001). (B) Cell viability was measured by FACS analysis after labeling with FITC-annexin V (FITC-A, X-axes) and propidium iodide (PI, Y-axes) of cells maintained 5 days at 25°C and then warmed-up at 37°C for 4h (4h) in the presence or in the absence of 15mM of NAC added at T0. (C) Percentage of apoptotic cells in the indicated culture conditions.

Heat shock has been shown to induce an oxidative stress in HEK293 cells [31]. Oxidative stress was analysed here during cold shock and rewarming. The level of ROS in cells kept at 25°C for 5 days was similar to that of control cells cultured at 37°C (not illustrated) and progressively increased after rewarming (plain grey bars in Figure 6A). It was significantly reduced by the ROS scavenger N-acetylcysteine (NAC) as expected (dashed bars in Figure 6A). As oxidative stress may lead to apoptosis, the effect of NAC on induction of apoptosis by cold shock and rewarming was investigated by FACS analysis. NAC, whether added at the beginning or at the end of the period in mild hypothermia, reduced cell death (compare Figure 6B to Figure 4A) at all time points (Figure 6C). Similar induction of ROS and reduction of cell apoptosis by NAC were observed in another cell line, namely MG63 cells (Figure S5, A, B and C).

**Figure 7:**
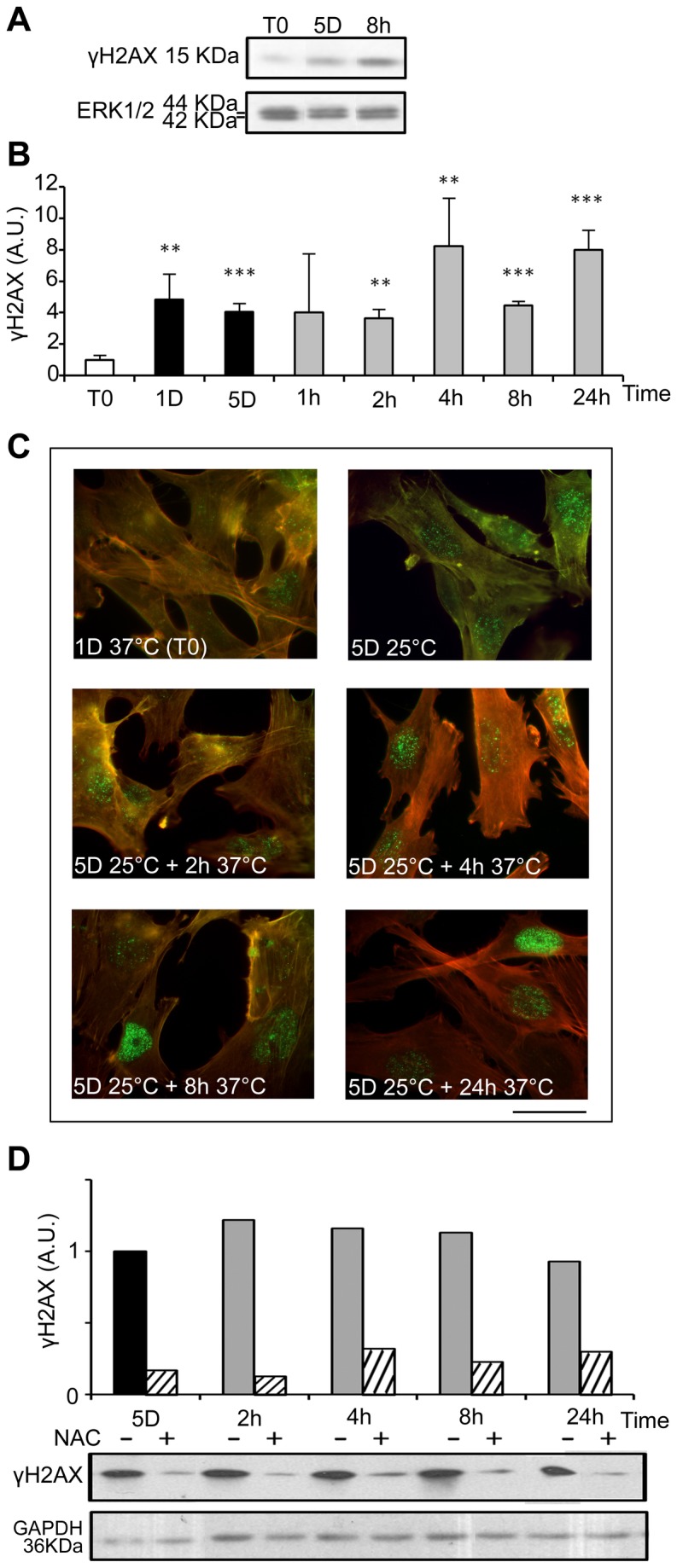
Cold shock and rewarming induce H2AX phosphorylation through ROS production. The levels of phosphorylated H2AX (γH2AX) were analyzed in WI26 cells cultured at 25°C for 1 and 5 days and then warmed-up at 37°C for 1 to 24h. (A) Representative western blots. (B) γH2AX was quantified by western blotting. Data are expressed in arbitrary units after normalization by ERK1/2, taking T0 as 1. (C) γH2AX in WI26 cells in normal conditions (1D37°C) and during cold shock for 5 days and rewarming for 2 to 24h was evidenced by immunostaining. Actin stress fibers are stained in red and γH2AX in green. Bar: 100µm. (D) γH2AX was measured by western blot in WI26 cells cultured at 25°C for 5 days and then warmed-up at 37°C for 2 to 24h in absence (-) and in presence (+) of NAC 15mM added at T0. After normalization by GAPDH, results are expressed in arbitrary units taking 5D as 1.

**Figure 8:**
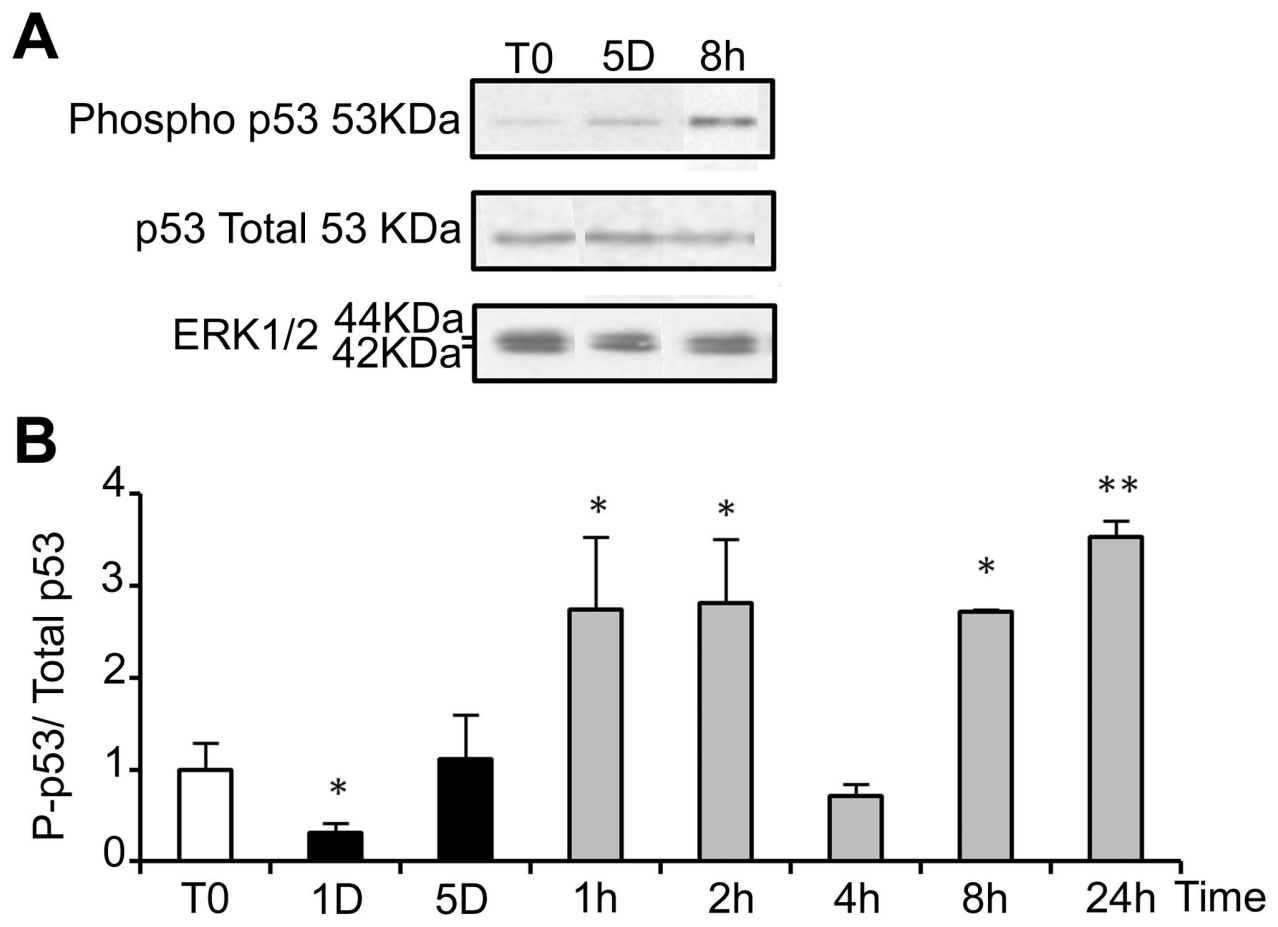
Return to normal temperature after a cold shock affects the phosphorylation of p53. Total and phosphorylated p53 (P-p53) were analyzed in WI26 cells cultured in normal conditions or at 25°C for 1 and 5 days and then warmed-up at 37°C for 1 to 24h. (A) Representative western blot. (B) Quantifications of the western-blots are expressed as the mean ratio P-p53/total p53, taking T0 as 1. Total protein loading was monitored by ERK1/2.

ROS are known inducers of oxidative DNA damage. To see if cold shock and rewarming may have the unexpected property to induce DNA damage, we analyzed the C-terminal phosphorylation of H2A histone family member X (H2AX), a double strand breaks tracer which participates in the DNA damage response and mediates DNA repair [32]. Mild hypothermia and rewarming induced a significant increase of H2AX phosphorylation (γH2AX) as shown by western-blot (Figure 7A and B) and immunostaining that showed an increased nuclear labeling (Figure 7C). A similar response was observed in osteoblastic cell lines (MG-63) and endothelial cells (HBME-1 and HMEC), showing that temperature-induced DNA damage is not restricted to WI26 cells (Figure S6). As illustrated in Figure 7D, γH2AX was strongly reduced upon NAC treatment, indicating a causal relationship between oxidative stress and DNA damage. We further investigated the phosphorylation of p53 and the expression of the alternative spliced VEGF111 variant [33], two processes known to be induced in response to DNA damage. Phospho-p53 signal was indeed significantly increased upon rewarming after 5 days of hypothermia (Figure 8). The expression of VEGF111, barely detectable after 5 days at 25°C, was strongly increased upon rewarming in WI26 cells (Figure S7, A and B). A similar induction was observed in other cell types of different lineage such as Hela cells, HMEC and MG63 (Figure S7, C), again underscoring the generalized cellular response.

## Discussion

Our work highlights deleterious effects on cell phenotype during mild hypothermia and return to normothermia. In agreement with others, we observed that hypothermia induces a re-programming of gene expression, RNA processing and intracellular signaling, and affects cell shape, growth and survival. Additionally, far from reducing the effects of cold shock, the return to physiological temperature (37°C) rather sustains or exacerbates some of them, in accordance with previous reports [1,3,11]. Rewarming also induces a burst of ROS, an effect likely induced by the sudden increase of mitochondrial electron transport [5].

General transcription and translation are reduced in hypothermia but remain able to proceed at a sufficient rate to ensure expression of at least some specific genes [2,34]. Hypothermia can also modulate the level and stabilization of specific transcripts as described for CIRBP [35]. In agreement with previous finding [5,11,36], the return to normothermia after a cold shock induced a heat shock-like response, as observed by the increased expression of HSP70 largely exceeding that of normothermic conditions during the first 8 hours of rewarming. This suggests that the sensing mechanism of heat shock detects a relative, instead of an absolute, hyperthermia.

It was reported that the proliferation rate of mammary cells significantly decreases with temperature [37] and human normal fibroblasts undergo a cell cycle arrest around 28°C. Our data only partly agree those of Matijasevic et al. The authors postulated that the cold-induced inhibition of cycle progression was linked to the activation of p53 [37]. Accordingly, we found that hypothermia resulted in a general inhibition of DNA synthesis of WI-26 cells. However, in our model, increased phosphorylation of p53 was not observed at 25°C. Furthermore, p53 deficient MG-63 cells, showed the same growth arrest at 25°C as cells proficient in p53 (data not shown). During rewarming, we systematically observed an early and transient increase of DNA content in WI26 and several other cell types (not shown). A similar “wave-patterned” [^3^H]-thymidine incorporation also occurred. In accordance with these data, we found that cells maintained at 25°C were arrested in G1 and G2 phase (data not shown) while only cells in G2 were observed within two hours of rewarming confirming a burst of DNA synthesis early after temperature transition. This observation is in agreement with previous reports [38,39] showing that cold-induced growth arrest might not only occur at a single check point. It is further supported by the pattern of ERK1/2 phosphorylation, known as a main regulator of the G1 to S transition [40]. However, this burst of DNA synthesis is transient and rapidly followed by a progressive loss of cells.

Divergent data have been described concerning the effects of mild hypothermia on apoptosis. Some studies showed induction of apoptosis upon cold shock while others did not or even suggested it had a protective effect against subsequent heat-induced cell death [41–43]. These discrepancies may be due to different experimental conditions in terms of treatment duration and/or range of temperatures. In our model, a low level of apoptosis and cell death was induced during hypothermia but sharply increased during rewarming, likely explaining the significant cell loss observed after two and three days of culture at 37°C.

Apoptosis and autophagy are triggered by both common and distinct signals [44–46]. In many circumstances, the two processes are mutually exclusive. Autophagy and Akt phosphorylation are commonly considered as pro-survival processes which counteract or delay apoptosis. However, at an overwhelming level of stress, massive autophagy can kill the cells, possibly by maintaining a significant level of ATP which may serve for the energy-consuming apoptotic processes [44]. Apoptosis and autophagy may therefore co-exist as it seems to be the case here. While autophagy has been observed in response to heat shock [14,47] or during the return to normothermia after cold-shock [48], we clearly showed here the induction of autophagy by hypothermia already after 1 day at 25°C. Activation of JNK was shown to be involved the induction of autophagy by different cell stressors [49]. However, as phosphorylation of JNK occurs only after warming-up in our model, the induction of autophagy in reaction to cold shock appeared to be JNK-independent. Several arguments such as cell cycle arrest and aberrant splicing of several molecules such as BAX, Bcl-X, FAS, VEGF, HDM-2, PIG-3 and caspase-9 (reported here and data not shown) suggested the intriguing possibility that DNA damage and a DDR are induced by cold shock and rewarming [33,50,51]. This was strongly supported by the increased phosphorylation of histone H2AX, a hallmark of DNA damage response (DDR), and that of p53 at least during rewarming. In agreement with other authors [52,53], we observed that rewarming of the cells induced an oxidative burst and we hypothesize that it may induce DNA damage, trigger the DDR and provoke apoptosis. According to this hypothesis, scavenging ROS by N-acetylcysteine significantly reduced apoptosis and cell death as well as γH2AX levels. These data strongly suggest a causative relationship between ROS production, induction of DDR, apoptosis and ultimately cell death in our model of mild hypothermia and rewarming. The mechanisms leading to DNA damage during the cold-shock phase remain however elusive.

The observation that hypothermia and, more importantly, rewarming induce an oxidative burst, apoptosis and a DDR might have practical consequences in biology and medicine and should be taken in consideration for cells, clinical samples or organs conservation and transportation prior to transplantation. They could also bring new insights for investigating adaptation to low temperatures and rewarming during therapeutic or accidental hypothermia.

## Supporting Information

Table S1 Sequences of the primers used for RT-PCR analyses. The number of PCR cycles and the size of the RT-PCR products are also indicated. 28S: ribosomal RNA 28S subunit (cellular: endogenous RNA; synth: synthetic control co-amplified with cellular 28S to monitor the efficiency of the reaction); BAX: BCL2-associated X protein; BCL-2: B-cell CLL/lymphoma 2; BCL-X: bcl-2-related gene (long - short: long and short isoforms of the gene); CIRBP: cold inducible RNA binding protein; HSP-70: heat shock protein 70; RBM3: RNA biding motif protein 3; VEGF: vascular endothelial growth factor.
(TIF)

Figure S1 Cold shock and rewarming affect markers of apoptosis. The expression of the anti-apoptotic Bcl-2 and pro-apoptotic BAX genes was quantified by RT-PCR in WI26 cells routinely maintained at 37°C or cultured at 25°C for 1 and 5 days before warming up at 37°C for 1h to 24h (abbreviations as in Figure 2). (A) Representative western blot showing the RT-PCR products. (B) Levels (mean ± SD) of Bcl-2, BAX mRNA and BAX to Bcl-2 ratio.
(TIF)

Figure S2 Cold shock and rewarming affect the splicing of Bcl-X. The expression of the Bcl-X gene was quantified by RT-PCR. (A) Representative example in WI26 cells routinely maintained at 37°C or cultured at 25°C for 1 to 5 days before warming up at 37°C for 1h to 24h. (B) Mean total Bcl-X mRNA, obtained by summing the signal of the two isoforms, is expressed in arbitrary units after normalization for the 28S mRNA, taking T0 as 1. (C) Mean Bcl-X_S/L_ ratio, taking T0 as 1.
(TIF)

Figure S3 Cold shock and rewarming affect Akt activation. Akt, phospho-Akt and ERK1/2 were quantified by western blot in WI26 cells cultured at 25°C for 1 and 5 days before warming up at 37°C for 1h to 24h. ERK1/2 was used to monitor equal protein loading. (A) Representative western Blot. (B) Results are expressed as the ratio (mean ± SD) of phospho-Akt (P-Akt)/ total Akt. T0 sample was arbitrary set at 1.
(TIF)

Figure S4 Cold shock and rewarming affect MG63 cells viability. Cells were cultured at 25°C for 5 days and then warmed-up at 37°C for 2 to 24h. Cells kept at 37°C were used as control (T0). Analysis was performed after cell labeling with FITC-annexin V (FITC-A, X-axes) and propidium iodide (PI, Y-axes) on 11.600 to 30.000 events collected for each experiment. (A) Examples of dots graphs (I) and annexin V curves (II) of control cells (T0) and cells maintained 5 days at 25°C and then warmed-up at 37°C for 4h (4h). Alive cells [A] (double negative staining), in early apoptosis [EA] (annexin V positive, PI negative), in late apoptosis [LA] (double positive) and necrotic [N] (annexin V negative, PI positive) are indicated on the graphs by using (II) Annexin V curves were used to define the gating allowing to discriminate the populations. (B) Quantification of cell death in MG63 cultured at 37°C (T0) or 25°C for 5 days and subsequent warming-up at 37°C for 2 to 24h.
(TIF)

Figure S5 Rewarming-induced ROS production and ROS-dependant apoptosis in MG63 cells. (A) ROS were measured in MG63 cells cultured at 25°C for 5 days and then warmed-up at 37°C for 2 to 24h in absence (-) and in presence (+) of 15mM of NAC. Cells kept at 37°C were used as control (T0). Data are expressed in arbitrary units taking control cells as 1. Significant inhibition by NAC is indicated (# # p<0.01; # # # p<0.001). (B) Cell viability was measured by FACS analysis after cell labeling with FITC-annexin V (FITC-A, X-axes) and propidium iodide (PI, Y-axes) of cells maintained 5 days at 25°C and then warmed-up at 37°C for 4h (4h) in the presence or in absence of 15mM of NAC added at T0 (compare with Fig. 4SA, 4 hours). (C) Percentage of dead cells in the indicated culture conditions.
(TIF)

Figure S6 Cold shock and rewarming induce H2AX phosphorylation in different cell types. H2AX phosphorylation was measured by western blot in HBME-1, HMEC and MG63 cells after 5 days at 25°C and warming up at 37°C for 1h and 2h. Cells kept at 37°C were used as control (T0). Data were normalized using GAPDH as calibrator. Results are expressed in arbitrary units taking control cells (T0) as 1 (n=1).
(TIF)

Figure S7 Cold shock and rewarming affect the splicing of VEGF pre-mRNA. The levels of VEGF variants and of the 28S rRNA were measured by RT-PCR in WI26, Hela, HMEC and MG63 cells cultured in the indicated conditions. (A) Representative gel showing the various VEGF splice variants in WI26. Arrow indicated the VEGF111. The 28S rRNA was used as calibrator. (B) Levels of the VEGF111 variant (mean ± SD) in WI26 cells cultured in the indicated conditions. VEGF111 level was expressed in % of total VEGF, taking T0 as 1. (C) Expression of VEGF111 in epithelial (Hela), endothelial (HMEC) and osteoblastic (MG63) cells after 5 days at 25°C and warming up at 37°C for 1h and 2h taking T0 as 1 (n=1).
(TIF)
