# Supplementary figures and images for: Effects of Mild Cold Shock (25°C) Followed by Warming Up at 37°C on the Cellular Stress Response
Source: PLoS One. 2013 Jul 23;8(7):e69687. doi: 10.1371/journal.pone.0069687 (PMC3720612; doi:10.1371/journal.pone.0069687)

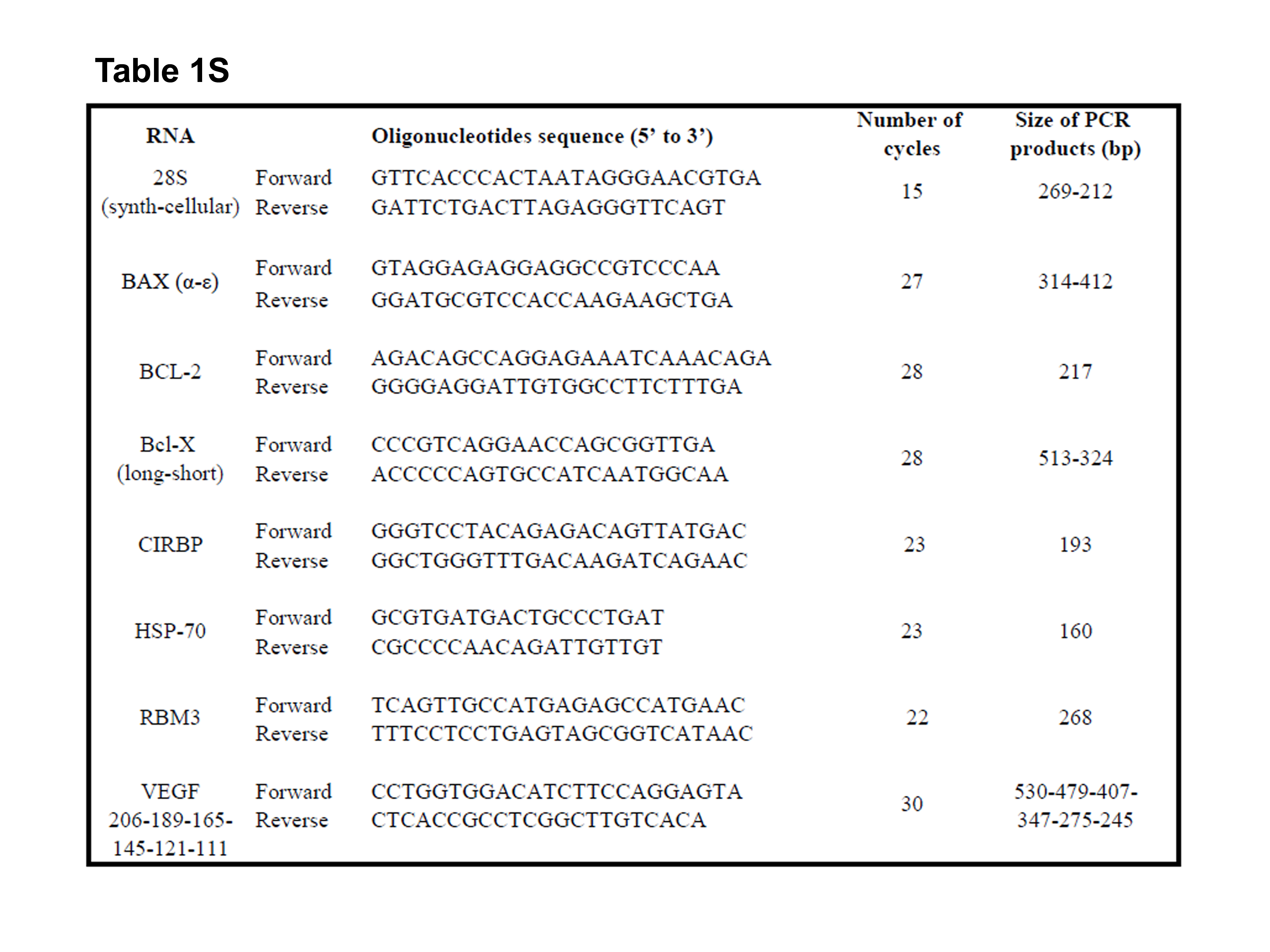

Supplement: Table S1 — The number of PCR cycles and the size of the RT-PCR products are also indicated. 28S: ribosomal RNA 28S subunit (cellular: endogenous RNA; synth: synthetic control co-amplified with cellular 28S to monitor the efficiency of the reaction); BAX: BCL2-associated X protein; BCL-2: B-cell CLL/lymphoma 2; BCL-X: bcl-2-related gene (long - short: long and short isoforms of the gene); CIRBP: cold inducible RNA binding protein; HSP-70: heat shock protein 70; RBM3: RNA biding motif protein 3; VEGF: vascular endothelial growth factor. (TIF) [file pone.0069687.s001.tif]

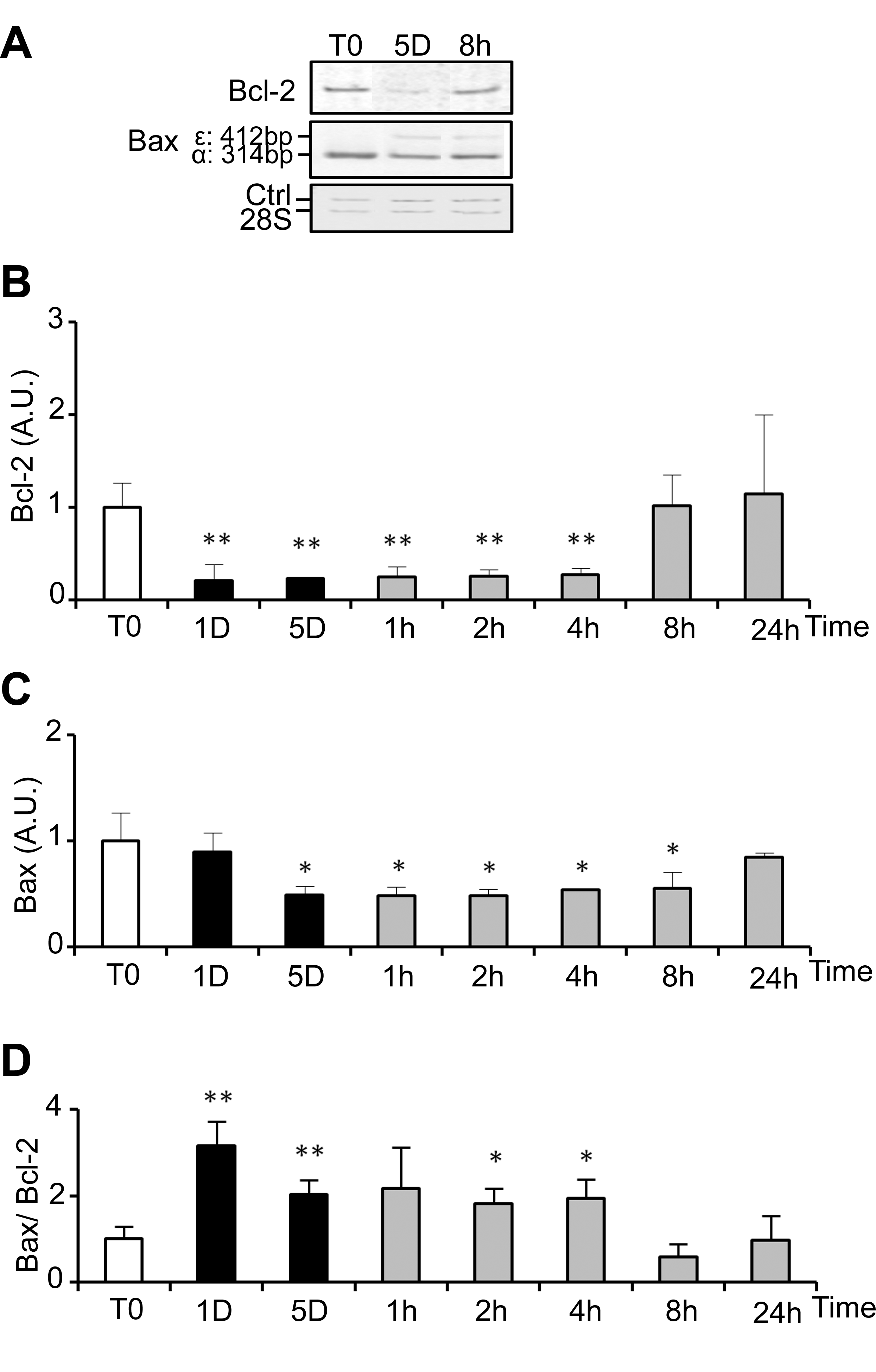

Supplement: Figure S1 — The expression of the anti-apoptotic Bcl-2 and pro-apoptotic BAX genes was quantified by RT-PCR in WI26 cells routinely maintained at 37°C or cultured at 25°C for 1 and 5 days before warming up at 37°C for 1h to 24h (abbreviations as in Figure 2). (A) Representative western blot showing the RT-PCR products. (B) Levels (mean ± SD) of Bcl-2, BAX mRNA and BAX to Bcl-2 ratio. (TIF) [file pone.0069687.s002.tif]

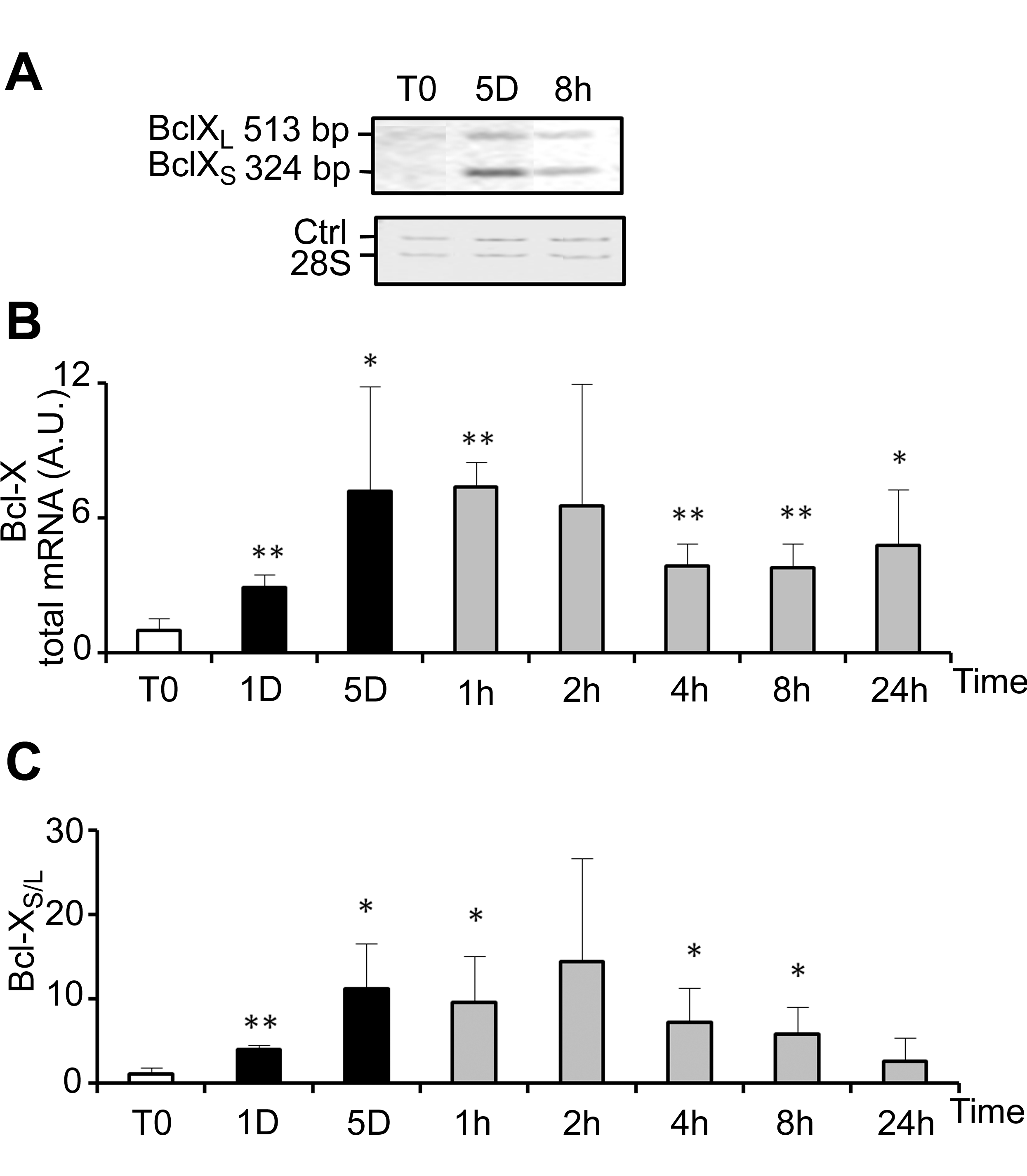

Supplement: Figure S2 — The expression of the Bcl-X gene was quantified by RT-PCR. (A) Representative example in WI26 cells routinely maintained at 37°C or cultured at 25°C for 1 to 5 days before warming up at 37°C for 1h to 24h. (B) Mean total Bcl-X mRNA, obtained by summing the signal of the two isoforms, is expressed in arbitrary units after normalization for the 28S mRNA, taking T0 as 1. (C) Mean Bcl-XS/L ratio, taking T0 as 1. (TIF) [file pone.0069687.s003.tif]

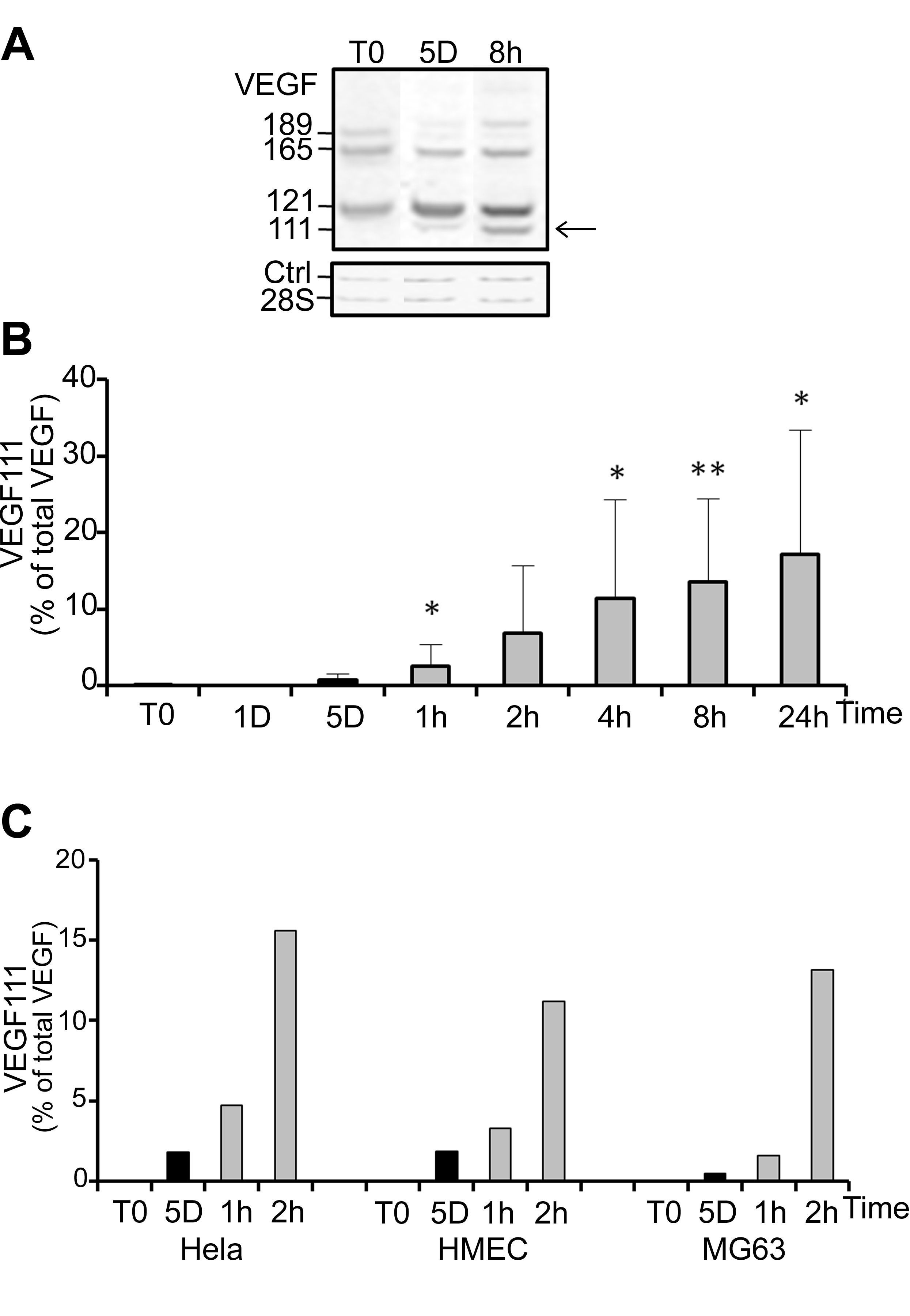

Supplement: Figure S3 — Akt, phospho-Akt and ERK1/2 were quantified by western blot in WI26 cells cultured at 25°C for 1 and 5 days before warming up at 37°C for 1h to 24h. ERK1/2 was used to monitor equal protein loading. (A) Representative western Blot. (B) Results are expressed as the ratio (mean ± SD) of phospho-Akt (P-Akt)/ total Akt. T0 sample was arbitrary set at 1. (TIF) [file pone.0069687.s004.tif]

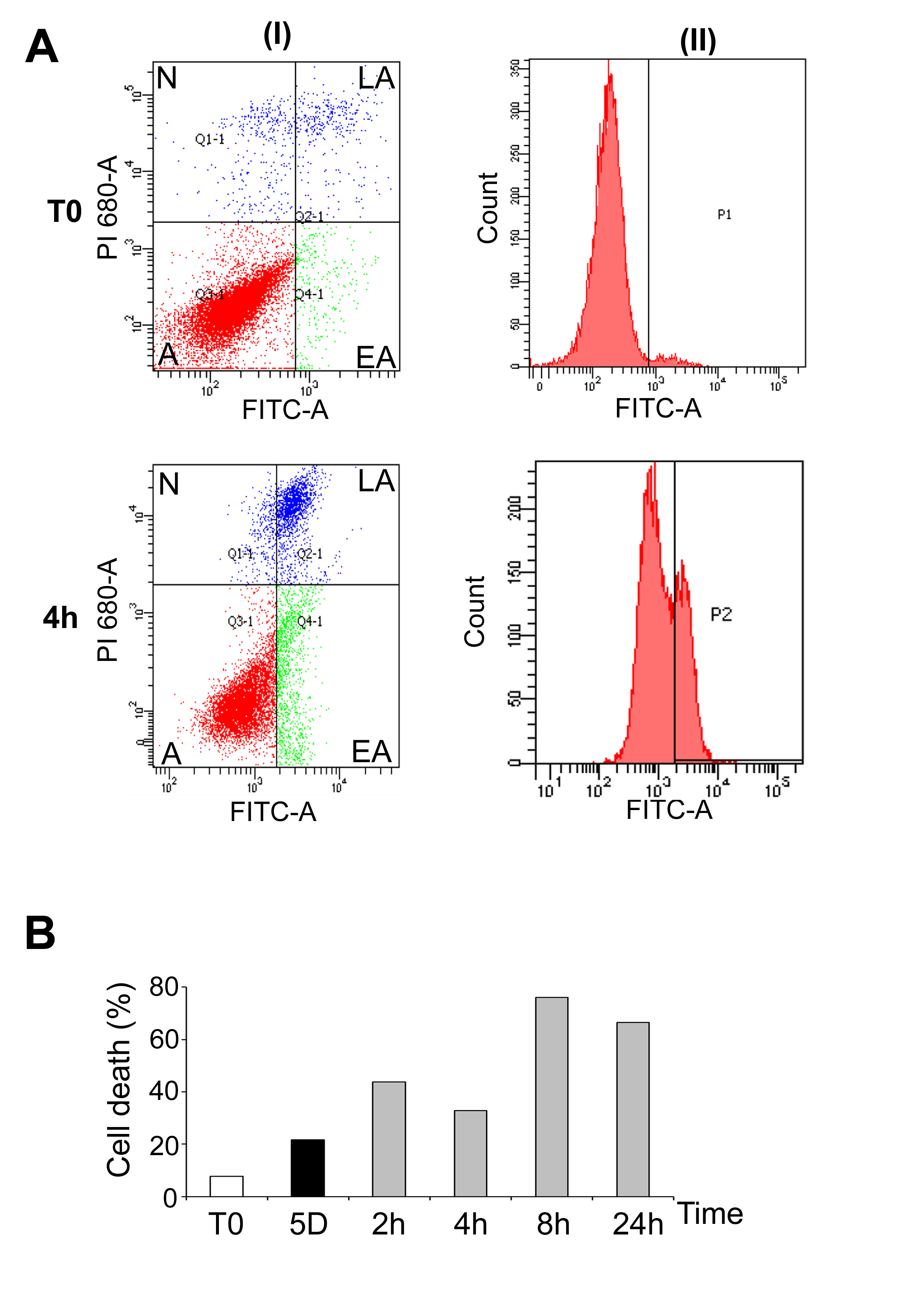

Supplement: Figure S4 — Cells were cultured at 25°C for 5 days and then warmed-up at 37°C for 2 to 24h. Cells kept at 37°C were used as control (T0). Analysis was performed after cell labeling with FITC-annexin V (FITC-A, X-axes) and propidium iodide (PI, Y-axes) on 11.600 to 30.000 events collected for each experiment. (A) Examples of dots graphs (I) and annexin V curves (II) of control cells (T0) and cells maintained 5 days at 25°C and then warmed-up at 37°C for 4h (4h). Alive cells [A] (double negative staining), in early apoptosis [EA] (annexin V positive, PI negative), in late apoptosis [LA] (double positive) and necrotic [N] (annexin V negative, PI positive) are indicated on the graphs by using (II) Annexin V curves were used to define the gating allowing to discriminate the populations. (B) Quantification of cell death in MG63 cultured at 37°C (T0) or 25°C for 5 days and subsequent warming-up at 37°C for 2 to 24h. (TIF) [file pone.0069687.s005.tif]

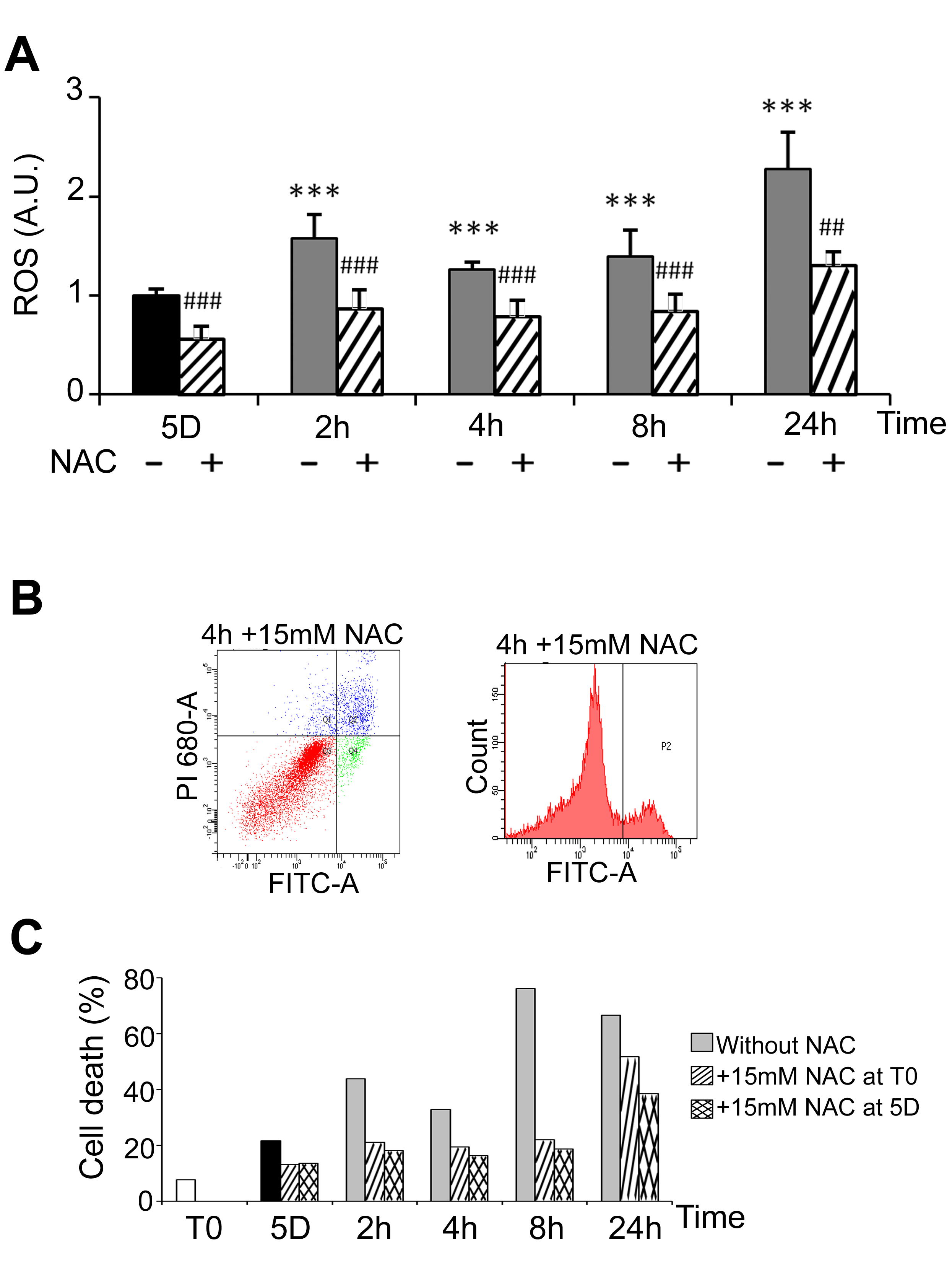

Supplement: Figure S5 — (A) ROS were measured in MG63 cells cultured at 25°C for 5 days and then warmed-up at 37°C for 2 to 24h in absence (-) and in presence (+) of 15mM of NAC. Cells kept at 37°C were used as control (T0). Data are expressed in arbitrary units taking control cells as 1. Significant inhibition by NAC is indicated (# # p<0.01; # # # p<0.001). (B) Cell viability was measured by FACS analysis after cell labeling with FITC-annexin V (FITC-A, X-axes) and propidium iodide (PI, Y-axes) of cells maintained 5 days at 25°C and then warmed-up at 37°C for 4h (4h) in the presence or in absence of 15mM of NAC added at T0 (compare with Fig. 4SA, 4 hours). (C) Percentage of dead cells in the indicated culture conditions. (TIF) [file pone.0069687.s006.tif]

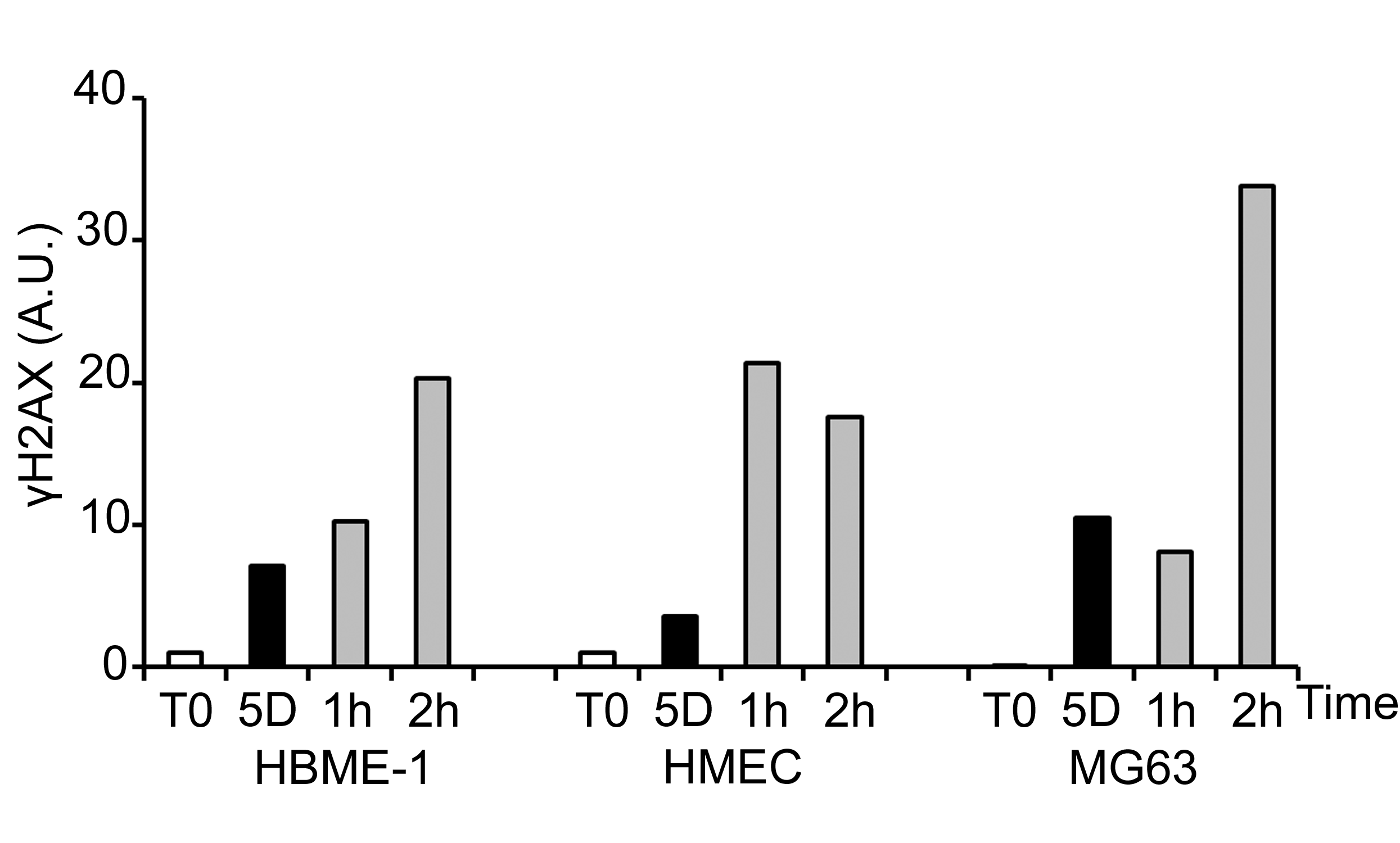

Supplement: Figure S6 — H2AX phosphorylation was measured by western blot in HBME-1, HMEC and MG63 cells after 5 days at 25°C and warming up at 37°C for 1h and 2h. Cells kept at 37°C were used as control (T0). Data were normalized using GAPDH as calibrator. Results are expressed in arbitrary units taking control cells (T0) as 1 (n=1). (TIF) [file pone.0069687.s007.tif]

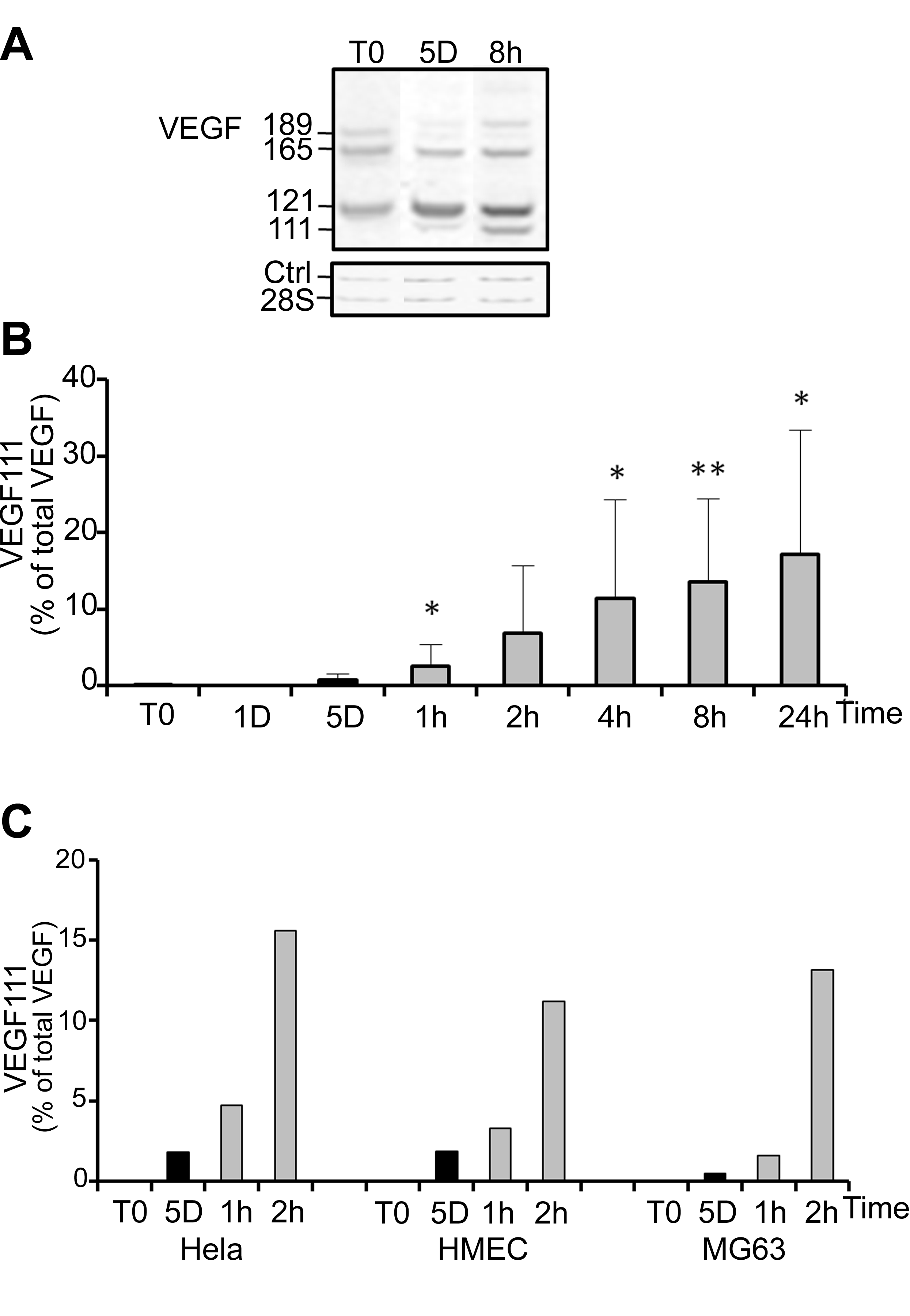

Supplement: Figure S7 — The levels of VEGF variants and of the 28S rRNA were measured by RT-PCR in WI26, Hela, HMEC and MG63 cells cultured in the indicated conditions. (A) Representative gel showing the various VEGF splice variants in WI26. Arrow indicated the VEGF111. The 28S rRNA was used as calibrator. (B) Levels of the VEGF111 variant (mean ± SD) in WI26 cells cultured in the indicated conditions. VEGF111 level was expressed in % of total VEGF, taking T0 as 1. (C) Expression of VEGF111 in epithelial (Hela), endothelial (HMEC) and osteoblastic (MG63) cells after 5 days at 25°C and warming up at 37°C for 1h and 2h taking T0 as 1 (n=1). (TIF) [file pone.0069687.s008.tif]
